# Aggregation of biologically important peptides and proteins: inhibition or acceleration depending on protein and metal ion concentrations

**DOI:** 10.1039/c9ra09350h

**Published:** 2019-12-24

**Authors:** Benjamin Gabriel Poulson, Kacper Szczepski, Joanna Izabela Lachowicz, Lukasz Jaremko, Abdul-Hamid Emwas, Mariusz Jaremko

**Affiliations:** Division of Biological and Environmental Sciences and Engineering (BESE), King Abdullah University of Science and Technology (KAUST) Thuwal 23955-6900 Saudi Arabia mariusz.jaremko@kaust.edu.sa lukasz.jaremko@kaust.edu.sa; Department of Medical Sciences and Public Health, University of Cagliari, Cittadella Universitaria 09042 Monserrato Italy; Core Labs, King Abdullah University of Science and Technology (KAUST) Thuwal 23955-6900 Saudi Arabia abdelhamid.emwas@kaust.edu.sa

## Abstract

The process of aggregation of proteins and peptides is dependent on the concentration of proteins, and the rate of aggregation can be altered by the presence of metal ions, but this dependence is not always a straightforward relationship. In general, aggregation does not occur under normal physiological conditions, yet it can be induced in the presence of certain metal ions. However, the extent of the influence of metal ion interactions on protein aggregation has not yet been fully comprehended. A consensus has thus been difficult to reach because the acceleration/inhibition of the aggregation of proteins in the presence of metal ions depends on several factors such as pH and the concentration of the aggregated proteins involved as well as metal concentration level of metal ions. Metal ions, like Cu^2+^, Zn^2+^, Pb^2+^*etc.* may either accelerate or inhibit aggregation simply because the experimental conditions affect the behavior of biomolecules. It is clear that understanding the relationship between metal ion concentration and protein aggregation will prove useful for future scientific applications. This review focuses on the dependence of the aggregation of selected important biomolecules (peptides and proteins) on metal ion concentrations. We review proteins that are prone to aggregation, the result of which can cause serious neurodegenerative disorders. Furthering our understanding of the relationship between metal ion concentration and protein aggregation will prove useful for future scientific applications, such as finding therapies for neurodegenerative diseases.

## Abstract 2

The process of aggregation of proteins and peptides is dependent on the concentration of proteins, and the rate of aggregation can be altered by the presence of metal ions, but this dependence is not always a straightforward relationship.

## Graphical abstract

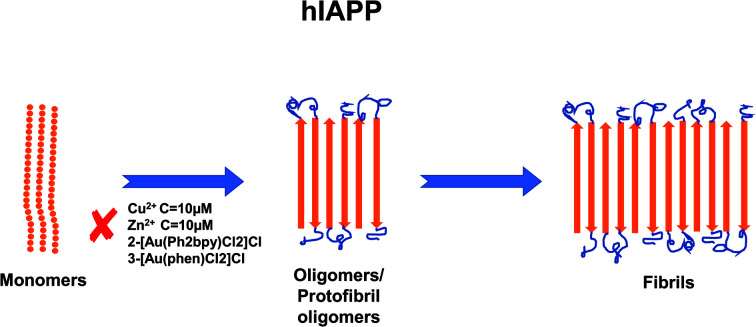

## 1. Introduction

The rate of aggregation of proteins depends strongly on the concentration of the aggregating proteins, but this relationship is not always straightforward.^1^ This dependence is also true for the most common protein in human blood, albumin (HSA, at concentrations of *ca.* 0.63 mM), which is a universal carrier of various substances in the blood of organisms, including metal ions in their complex forms.^2^ HSA aggregation, which normally does not occur under physiological conditions, is induced by the presence of metal ions such as Co^2+^, Cr^3+^ and Ni^2+^ (with a metal ion ratio up to 1 : 8 at pH = 7.3), with Cr^3+^ promoting the strongest aggregation rate.^3^ Metal ions like Cu^2+^ participate in pathological transformations that lead to aggregation, such as prion (PrP^C^) proteins for example, which bind to tandem octapeptide repeats,^4–6^ leading to numerous severe neurological pathologies.^7–10^ Recently, some authors have postulated that on the molecular level, the N-terminal domain of PrP^C^ may act as a toxic effector whose activity is normally auto-inhibited by metal ion-assisted intramolecular association with the C-terminal domain.^6^ Therefore, it should be pointed out that at the higher concentrations of Cu^2+^ ion, the individual tandem repeats are able to coordinate with different geometries up to a total of four Cu^2+^ ions, mainly by imidazole rings of histidine, together with the amide nitrogen of these residues,^6,11^ as well as most likely by tryptophan side-chains,^4^ preventing the PrP molecule from misfolding into the pathological PrP^C^ form^4,11,12^ with weaker micromolar affinity,^6^ suggesting that the influence of the Cu^2+^ ions on the transformation of the prion protein into its pathological forms depends on the concentration of their free accessible form in solution.^6^ On top of that, it is still not known, if PrP binds Cu^2+^ ions within the positive or negative cooperativity effects.^11^

**Scheme 1:**
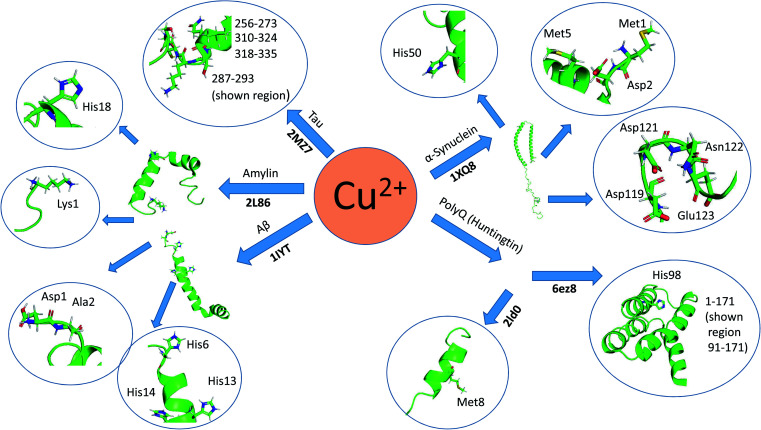
Graphical representation of residual binding sites of Cu^2+^ and their respective proteins. (Bolded text represents the PDB IDs of the proteins).

**Scheme 2:**
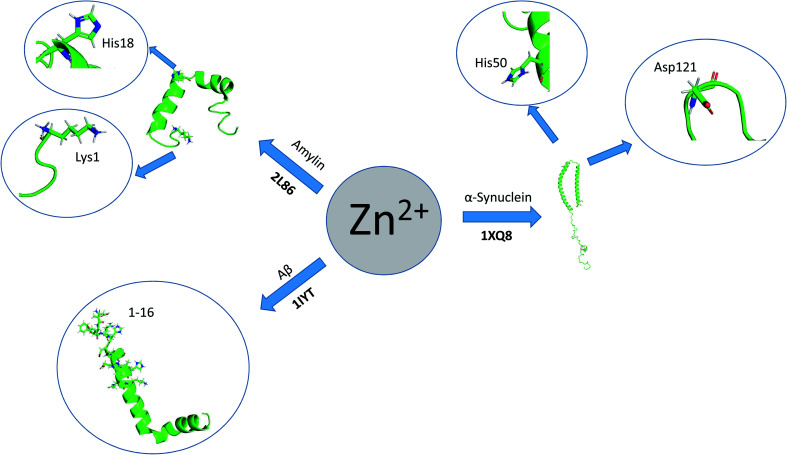
Graphical representation of residual binding sites of Zn^2+^ and their respective proteins. (Bolded text represents the PDB IDs of the proteins).

Here, we review a number of biomolecules whose aggregation rates are dependent on their concentration and metal ion coordination properties. The biomolecules reviewed are the following: islet amyloid polypeptide (IAPP), which contributes to glycemic control and has implications for Type II diabetes,^13,14^ Aβ peptide and Tau protein, which are the main components of amyloid deposits found within the neuronal cells of patients with Alzheimer's disease (AD).^15,16^ α-Synuclein, which is strongly associated with Parkinson's disease (PD).^17^Table 1 lists select metal ions and their binding sites to the proteins discussed in this review. Schemes 1 and 2 give a visual representation of these binding sites.

**Table 1:** Summary of metal ions and binding sites to proteins of interest

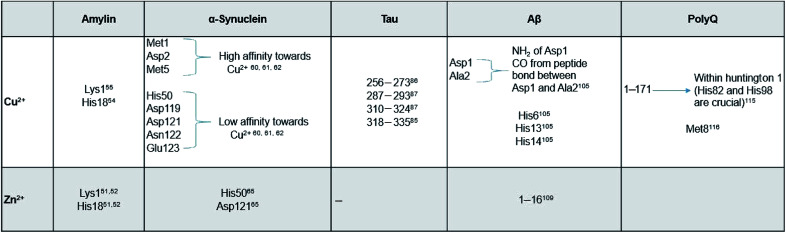

## 2. General conditions of peptide aggregation

There are more than 20 amyloid diseases‡‡Alzheimer's disease; spongiform encephalopathies; Parkinson's disease; primary systemic amyloidosis; secondary systemic amyloidosis; Fronto-temporal dementias; senile systemic amyloidosis; familial amyloid polyneuropathy; hereditary cerebral amyloid angiopathy; haemodialysis-related amyloidosis; familial amyloid polyneuropathy; Finnish hereditary systemic amyloidosis; Type II diabetes; medullary carcinoma of the thyroid; atrial amyloidosis; hereditary non-neuropathic systemic amyloidosis; injection-localised amyloidosis; hereditary renal amyloidosis; amyotrophic lateral sclerosis; Huntington's disease; spinal and bulbar muscular atrophy; spinocerebellar ataxias; spinocerebellar ataxia. characterized by the deposition of amyloid fibrils and plaques in central nervous system (CNS) and in some peripheral tissues.^18^ Moreover, there are other misfolding/conformational pathologies (*e.g.* cystic fibrosis, Marfan syndrome, amyotrophic lateral sclerosis), featured by the presence of “wrongly” folded proteins (with respect to non-pathological conditions).^19^ Also, in some cancer cells, certain proteins have “incorrect” structure. Surprisingly, amyloid fibrils and plaques are more toxic at the early stages of polymerization rather than the final product.^18^

At the beginning the protein aggregates are soluble, but gradually become insoluble when they exceed solubility limits. Protein–protein interactions in the aggregates can be electrostatic and/or hydrophobic and can lead to minor conformational changes. Lowering the surface charge of protein can increase aggregation. Most aggregation processes are nucleation-dependent.^20^

The primary amino acid sequence of proteins is an inherent feature of aggregation processes.^21^ In many aggregation processes, the initial reaction is the formation or exchange of intermolecular disulfide bond.^22^ Cysteines located on the protein surface are more easily involved in the aggregation than cysteine residues in the inert part. The disulphide bond aggregation of human serum albumin was studied by Wetzel *et al.*, (1980) who showed that unfolding of the pocket containing the free –SH group of cysteine-34 prevent the formation of disulphide bridges and leads to stable aggregates and irreversible structural alterations.^23^

Amyloids share common structure (high β-sheet content)^24^ and the aggregation process occurs in the extracellular space of the CNS (*e.g.* Alzheimer's and Creutzfeldt–Jakob diseases), and some peripheral tissues and organs (*e.g.* liver, heart and spleen-systemic amyloidosis and type II diabetes).^25,26^ Primary or secondary amyloidosis, can also be found in skeletal tissue and joints (*e.g.* haemodialysis-related amyloidosis) and in some organs (*e.g.* heart and kidney). Surprisingly, the plaques' formation is less frequent in peripheral nervous system.

Up to know it is not well established, whether protein aggregation is the cause or consequence of the pathologies. Moreover, early amyloid plaques are similar structurally to pores made of bacterial toxins and pore-forming eukaryotic proteins, which suggests the functional significance of such plaque constructions.^18^

Aggregation occurs when the normal protein folding machinery does not work correctly. Such black out can be caused by specific mutations, which enhanced protein synthesis or reduced their clearance. Molecular chaperones that process the protein degradation prevents pathologies in normally functioning organisms. Different degenerative diseases have been associated with deterioration of the ubiquitin-proteasome pathway (Alzheimer's disease, Fronto-temporal dementia, Parkinson's disease, dementia with Lewy body, amyotrophic lateral sclerosis, poly-Q extension disorders, Huntington's disease, spinocerebellar ataxias, spinobulbar muscular atrophy).^27^ It was also shown that 30–33% macromolecular crowding, which can be a result of ageing^28^ or of progression through the cell cycle,^29^ can lead to higher molecular binding affinities.^30^ Amyloid diseases are manifest most frequently late in lifespan, when aging leads to DNA methylation. It could be deduced that DNA changes lead to up-regulation of the expression of some proteins, which in turn accumulate and aggregate inside cells.^18^

**Scheme 3:**
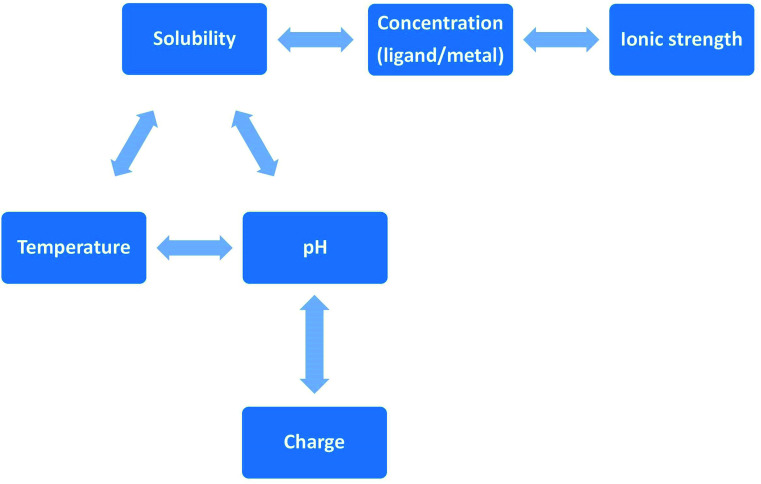
Direct and indirect correlation of environmental factors that influence peptides' aggregation. The solubility of a given solute in a given solvent typically depends on temperature. Depending on the nature of the solute the solubility may increase or decrease with temperature. For most solids and liquids, their solubility increases with temperature. Ionic compounds have limited water solubility, and the amount of soluble products is defined by the solubility product (*K*_sp_). This value depends on the type of salt, temperature, and the common ion effect. *K*_sp_ depends directly on ions activity, which is related to the activity coefficient and ion concentration. The pH–solubility profile of a weak acid or base is shown to be a function of its p*K*_sp_, and p*K*_a_, and uncharged species solubility and was widely described by Streng *et al.* (1984).^34^

More often protein aggregation is a result of wrong interactions with metal ions, local changes in environmental conditions (*e.g.* pH, temperature, ionic strength) (Scheme 3) or chemical modification (oxidation, proteolysis). There are five main environmental conditions that influence the aggregation process, and they are directly (temperature and pH) or indirectly (pH and concentration) correlated. It was shown in the experimental studies that even small variation of environmental factors can significantly change the final results. Jha *et al.*, (2014) demonstrated that the amylin fibrillization is directly related to the pH, which is physiologically important.^31^

Also the final structure of plaques depends on the environmental conditions.^32^ The pH determines the type and the density of surface charge and the degree of protein structural disruption. Moreover, pH affects intramolecular folding and protein–protein interactions.^20^ Protein concentration is another important factor in aggregation process, while enhancing protein association or lead to the protein precipitation when it exceeds solubility limit. It is noteworthy that the sequence of the peptide affects its propensity to form or not amyloid structures under specific conditions: aggregation through unfolding intermediates and unfolded states (*e.g.* protein translocation through the membranes); or aggregation through protein self-association.^20^ Partially unfolded peptides exhibit hydrophobic sequences and have higher elasticity with respect to the folded state, thus have enhanced susceptibility to aggregation process.^33^

Bearing in mind arguments described above, it is necessary to conduct the *in vitro* experiments in the conditions similar as much as possible to that in the physiological conditions.

## 3. Islet amyloid poly-peptide (hIAPP), amylin

Islet amyloid polypeptide (IAPP) is a specific protein hormone consisting of 37 amino acids (3.9 kDa) in its native form, with the C-terminus amidated, and with a disulfide bridge between Cys-2 and Cys-7. IAPP is secreted from β-cells of the pancreas into the blood along with insulin. Amylin is a primary hormone that regulates and maintains blood glucose levels in the body, and its effects are complementary to insulin.^35,36^ Human IAPP (hIAPP) plays an active role in glycemic regulation by slowing gastric emptying and promoting satiety, thereby preventing postprandial spikes in blood glucose levels. However, it cannot be used as a drug for the treatment of diabetes because of its tendency to mis-fold and subsequently aggregate, resulting in the formation of cytotoxic fibrils,^13,37^ which are strongly associated with β-cell degeneration in Type 2 Diabetes Mellitus (T2DM).^38^ The rate of hIAPP aggregation depends on many factors that we discuss below.

It has been reported that His18 acts as an electrostatic switch that inhibits fibrillization (aggregation) in its charged state and is heavily pH-dependent.^31^ Modulations are observed even in the narrow physiological range of pH of 7.35–7.45.^39^ This relationship was clearly demonstrated by the usage of ThT dyes for monitoring hIAPP aggregation at different pH, related to the activity of H_3_O^+^ ions in solution that is directly related to their concentration in solution.

hIAPP is closely related with cytotoxicity, which heavily depends on its concentration as well as on how the “synthetic” peptide sample is prepared. The highest observed cytotoxic potentials of hIAPP is at concentrations of 25 μM for full length hIAPP, and 40 μM for the 8–37 hIAPP fragment.^40^ The range of reported cytotoxicity for hIAPP, expressed as a percentage of dead cells, is believed to be from 15 to 80% for exposure to 5–25 μM of hIAPP for a duration of 24–48 h.^41^

**Fig. 1:**
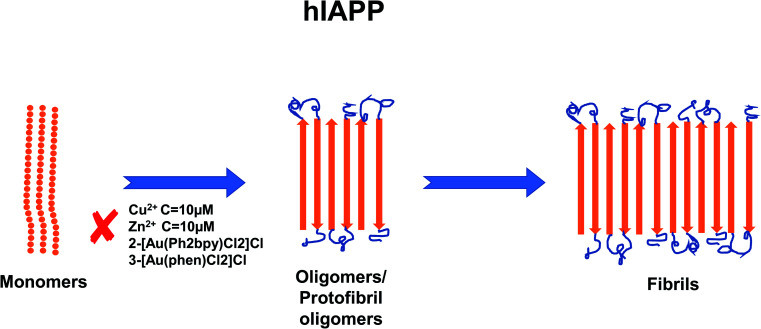
Schematic representation of metal ion concentration-dependent inhibition or acceleration of hIAPP aggregation. Zn^2+^ (10 μM), Au^3+^ and Cu^2+^ (10 μM) inhibit the formation of aggregates. The figure has been copied and adapted with permission from Alghrably *et al.*, (2019).^14^

**Fig. 2:**
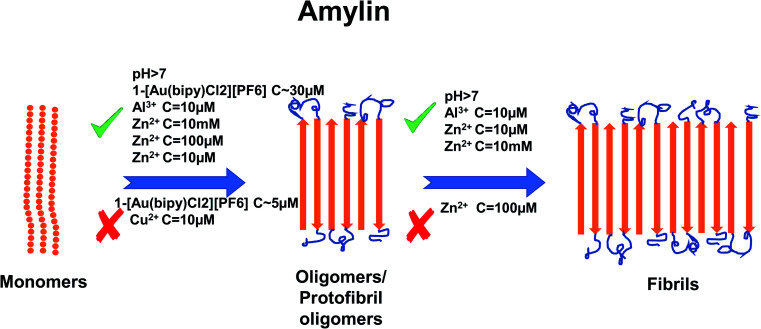
Schematic representation of metal ion concentration-dependent inhibition or acceleration of amylin aggregation. Zn^2+^ (10 μM, 10 mM), Al^3+^ (10 μM) and Au^3+^ (∼30 μM) promote the formation of aggregates, while Cu^2+^ (10 μM) and Au^3+^ (∼5 μM) inhibit aggregate formation. Zn^2+^ (100 μM) promotes the formation of oligomers but inhibits the formation of fibrils. The figure has been copied and adapted with permission from Alghrably *et al.*, (2019).^14^

In recent years, the importance of the role of the metal ions Cu^2+^, Zn^2+^, Al^3+^ and Fe^2+^/Fe^3+^ in the aggregation of hIAPP has been identified (see Fig. 1).^42,43^ In addition, their ability to modulate the proteolytic activity of hIAPP-degrading enzymes has been extensively studied.^14,44–46^ It was reported that, Zn^2+^ plays an important role in glycemic regulation, which is reflected in their high concentrations in the interior of dense granule cores ranging from 10 to 20 mM, confirming their physiological importance.^47–50^ The effect of concentrations of Zn^2+^ on hIAPP aggregation has been studied in detail. Several studies have shown that varying concentrations of Zn^2+^ have different effects on hIAPP aggregation and the different stages of the aggregation process. At high concentrations (10 mM) and in the early stages of aggregation (40 min), Zn^2+^ promote the formation of large Zn^2+^–amylin aggregates. In general, it has been reported that Zn^2+^ ion binds to amylin at the imidazole ring of His18 and the amine group of Lys1.^51,52^ At low Zn^2+^ concentrations (100 μM) and in the early stages of aggregation (40 min), Zn^2+^ induces the formation of even larger Zn^2+^–amylin aggregates than those formed at high concentrations of Zn^2+^. During the final stages of aggregation (when the amylin fibrils are formed), fiber formation is inhibited at low concentrations of Zn^2+^ and accelerated at higher concentrations.^14,53^ These findings have been supplemented by a study on the effect of Al^3+^, Fe^3+^, Zn^2+^ and Cu^2+^ at near physiological concentrations (10 μM, *i.e.*, in stoichiometric excess) on amylin at 0.4 and 2 μM (see Fig. 2).^42^ Cu^2+^ efficiently inhibited amylin aggregation at certain concentrations. Other studies report that Cu^2+^ binds to amylin at the imidazole ring of His18 (ref. 54) and to the three preceding amides at the N-terminal side of His18 (ref. 52) and at Lys1.^55^ An opposite effect was observed for Al^3+^ and Zn^2+^ at the same concentration levels. Fe^3+^ appeared to have very little influence on amylin aggregation for the metal ion and peptide concentration ranges that were tested. Further tests in the same study using sub-stoichiometric concentrations of the metal ions confirmed the inhibitive properties of Cu^2+^, to a lesser extent for Zn^2+^, and no influence of Al^3+^ on hIAPP aggregation.^43^ A recent study applied several experimental techniques such as ThT fluorescence and Atomic Force Microscopy (AFM) to examine different characteristic changes of hIAPP, and Dynamic Light Scattering (DLS) analysis was used to determine the particular effects of Au^3+^ complexes on the aggregation of hIAPP.^56^ Electrospray Ionization-Mass Spectrometry (ESI-MS) and the intrinsic fluorescence method were employed to investigate the binding properties between the Au complexes and hIAPP. He *et al.* (2015) used NMR spectroscopy to discover that complexes 2-[Au(Ph_2_bpy)Cl_2_]Cl (Ph_2_bpy = 4,4′-diphenyl-2,2′-bipyridyl) and 3-[Au(phen)Cl_2_]Cl (phen = 1,10-phenanthroline) strongly inhibited the aggregation of hIAPP, compared to complex 1-[Au(bipy)Cl_2_][PF_6_] (bipy = 2,2′-bipyridine), which promoted the formation of amylin oligomers/protofibrils at high concentrations (∼30 μM).^56^ However, at low concentrations (∼5 μM), it inhibited amylin oligomer formation, verifying the concentration dependence of the inhibition process.

Limited reported results in the scientific literature highlight an urgent need for a systematic and accurate study on the dependence of hIAPP aggregation on peptide and metal ions concentrations, with a particular emphasis on physiological conditions and concentration ranges.

## 4. α-Synuclein

α-Synuclein is a protein that consists of 140 amino acids and is present in large quantities in the brain.^57^ α-Synuclein is located within three domains: N-terminal lipid-binding α-helix, amyloid-binding central domain (NAC), and C-terminal acidic tail.^58^ In the human body, α-synuclein functions as a molecular chaperone for forming SNARE complexes (SNARE is a group of proteins that catalyzes the fusion of membranes in vesicle transport) in synapses, enables the release of neurotransmitters and regulates levels of glucose and the biosynthesis of dopamine.^58^ α-Synuclein has been identified as the main component of Lewy bodies – aggregates of protein characteristic to Parkinson's disease and other synucleinopathy diseases.^59,60^ The formation of aggregates of α-synuclein depends on factors such as pH, post translational modifications (PTM), polyamines and concentration of α-synuclein.^61^

Buell *et al.* (2014) found that the multiplication rate of α-synuclein is suppressed under neutral pH and inert conditions.^62^ However, changing the pH to mildly acidic (4.8–5.6 pH), *i.e.*, non-physiological pH, strongly affects the multiplication process, with the biggest impact at pH 5.2. Compared to the fibril elongation constant by monomer addition (2 × 10^3^ M^−1^ s^−1^ for PBS buffer), an acidic environment increases the fibril elongation rate constant by one order of magnitude and the rate of production of new fibrils (by secondary nucleation) increases by four orders of magnitude.^62^ Additionally, it was demonstrated that in physiological salt concentrations (150 mM NaCl), α-synuclein tends to form aggregates that can subsequently form gels.^62^

Another factor favoring the aggregation process is the initial concentration of α-synuclein.^63^ Uversky *et al.*, (2001) measured the change of ThT fluorescence intensity for various concentrations of α-synuclein: 21 μM, 70 μM, 105 μM and 190 μM. They found that the fluorescence intensity increased with higher concentrations of proteins, which demonstrates an increase in the α-synuclein aggregation rate in the form of fibrillation. Nonetheless, the concentration of 21 μM of α-synuclein was enough to start the fibrillation process.^63^

**Fig. 3:**
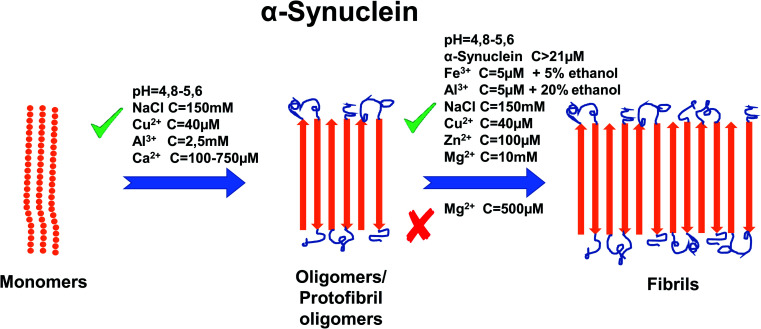
Schematic representation of metal ion concentration-dependent inhibition or acceleration of α-synuclein aggregation. Concentration of α-synuclein (more than 21 μM), Zn^2+^ (100 μM), Al^3+^ (2.5 mM, 5 μM in 20% ethanol), Cu^2+^ (40 μM), NaCl (150 mM), pH (4.8–5.6), Ca^2+^ (100–750 μM) and Fe^3+^ (5 μM in 5% ethanol) promote forming aggregates, whereas Mg^2+^, depending on concentration, inhibits aggregation (500 μM) or promotes (10 mM) formation of fibrils. The figure has been copied and adapted with permission from Alghrably *et al.* (2019).^14^

Metal ions such as Cu^2+^, Zn^2+^, Al^3+^, Fe^3+^, Ca^2+^ and Mg^2+^ have also been shown to affect aggregation rates.^64^ For copper, it has been shown that the addition of 40 μM of Cu^2+^ accelerates the aggregation rate by promoting the nucleation process of α-synuclein.^65^ In addition, Cu-induced fibrils have been shown to have the same morphology as those formed in the absence of Cu^2+^.^65^ There are two regions where Cu^2+^ binds to α-synuclein. One of them is located at N-terminal site with residues Met1, Asp2, Met5 that have high affinity to copper and residue His50 with low affinity. The other region is at C-terminal part with residues Asp119, Asp121, Asn122, Glu123 and binds copper ions with low affinity.^65–67^ For His50, the ability to bind Cu^2+^ is greatly affected by pH. It was shown that lowering the pH to the acidic values cease the ability of His50 to bind copper.^68^ Additionally the acetylation on N-terminal region of α-synuclein abolished its ability to bind Cu^2+^ at residue Met1, leaving His50 ability intact in this region.^66,69^ However, a recent paper^67^ shows that copper does not bind to His50 in α-synuclein fibrils. Instead, during the fibrillation process Cu^2+^ has the ability to bind to other residues in N-terminal and C-terminal sites and can “bounce” between them. Zn^2+^ at concentrations of 100 μM has been proven as an effective promoter of α-synuclein aggregation and specifically α-synuclein fibrillation *in vitro*.^70^ It has been proven that Zn^2+^ binds to residues His 50 with much lower affinity that in case of Cu^2+^ and Asp121 with similar affinity compared to Cu^2+^.^71^ Data shows that the addition of Al^3+^ to a high concentration of α-synuclein induces the formation of oligomers. Addition of 2.5 mM of AlCl_3_ shortened the time of fibril formation ∼3-fold and increased the rate of fibril formation ∼1.5 fold^63^ and these fibrils form structure similar in look to twisted ribbons. On the other hand, Fe^3+^ (5 μM) has been proven to promote α-synuclein aggregation but only when added in the presence of intermediate concentrations of ethanol (∼5%).^72^ In the same paper, it was also shown that Al^3+^ (5 μM) promotes aggregation in 20% ethanol but has a lesser effect on aggregation than Fe^3+^. Like for most divalent metals, binding site for Fe^3+^ is postulated to be in C-terminal region, possibly residue Asp121.^73^ A study from Nath *et al.* (2011) demonstrated that aggregation is also very dependent on Ca^2+^ concentrations, whereby higher concentrations of Ca^2+^ (from 100 μM to 750 μM) resulted in fewer monomers remaining in the sample because of the formation of aggregates.^74^ However, the concentration of Ca^2+^ required to induce α-synuclein aggregation in free solution is far higher than that required in order to induce aggregation at a hydrophobic glass surface.^74^ For a binding site of Ca^2+^, study shows that Ca^2+^ binds to the C-terminal domain (126–140) however, currently there is no information which particular residue is involved in the binding process.^74^ There is currently a lack of information about the effect of Pb on aggregation *in vitro*, although it has been demonstrated that Zn^2+^, Al^3+^ and Pb^2+^ enable methionine-oxidized α-synuclein,^75^ to form aggregates at the same rate as the non-oxidized α-synuclein.^76^ The interaction effects of Mg^2+^ ions with α-synuclein aggregation have not been investigated we well as Zn and Cu ions hence further investigations are necessary. One study showed that Mg^2+^ at 500 μM has the ability to inhibit the aggregation process of α-synuclein (23 μM), even under the iron-induced aggregation (50 μM of Fe^3+^ on 8 μM of α-synuclein).^77^ On the other hand, Hoyer *et al.* (2002) have shown that 10 mM of Mg^2+^ (at pH 7.0) helps to form aggregates composed of densely packed short fibrillary elements.^78^ For a summary of the effects of metal ion concentration on α-synuclein, refer to Fig. 3.

In conclusion, the evidence shows that metal ions can inhibit or accelerate the aggregation of α-synuclein. Nevertheless, much work remains to be done in order to gather and analyze information on these effects. Our brief literature review indicates a fundamental need for further systematic research on concentration-dependent aggregation of proteins and the influence of metal ions on the aggregation process.

## 5. Tau protein

The aggregation of Tau protein (TP) in neuronal cells is characteristic of Alzheimer's disease (AD).^79^ Although there is a clear correlation between the aggregation of TP and the progress of AD,^80^ the relationship between them still remains elusive, and several scientists are seeking methods to accurately model the exact relationship between them.^81,82^

TP is primarily responsible for stabilizing microtubules in neuronal cells. One of the mechanisms in which TP regulates the stability of these microtubules is *via* phosphorylation,^83,84^ though the exact association between TP and microtubules is not completely clear.^84,85^ Out of the 441 amino acids in Tau's peptide sequence (htau 40 human isomorph), 85 of them are phosphorylation sites. These phosphorylation sites are regulated both by kinase and phosphatase enzymes. A typical TP will have approximately 30 of its 85 phosphorylation sites phosphorylated.^86^ An abnormal TP will normally contain three times as much phosphate as a normal TP, at which point the TP is “hyperphosphorylated”. In its hyperphosphorylated state, TP cannot properly stabilize microtubules in neuronal cells, and aggregation of TP begins.^79^

Several studies have reported the effects of metal ion concentrations on TP aggregation, although many have reported contradictory results.^87^ For example, the mechanism of action of different metal ions are not consistent.^88^ The consensus, however, is that the higher the concentration of metal ions present in the brain, the more protein aggregation occurs, supporting the progress of AD. Below we discuss the impact of Cu^2+^, Zn^2+^ and Li^+^, as each shows acceleration or inhibition of TP.

The scientific literature shows that Cu^2+^ accelerates the aggregation of TP either by activation of GSK3β kinase^89^ or activation of CDK5.^90^ Voss *et al.* (2014) reported acceleration of TP aggregation with concentrations of 400 μM of Cu^2+^,^89^ whereas Crouch *et al.* (2009) reported acceleration of TP aggregation under concentrations of Cu^2+^ of 25 μM.^90^ These numbers seem reasonable, as Cu^2+^ typically has a concentration of about 10 μM at neuronal synapses, and at this concentration, TP aggregation does not normally occur.^91^

The literature regarding the precise binding site of Cu^2+^ is ambiguous, as authors report different binding sites. For example, one paper claims the binding site for Tau protein to be residues 318–335. By binding at this region of the TP, Cu^2+^ induces fibrillization *via* formation of alpha helices.^92^ However, Zhou *et al.* (2017) claim that Cu^2+^ simply modulates the aggregation of TP by binding it at residues 256–273 of the htau 441 isoform, and associates it with His-268.^93^ Still, Soragni *et al.* (2008) claim that Cu^2+^ has a minor impact on TP aggregation *in vitro*, and only binds to TP with micromolar affinity (approximately 0.5 μM).^94^ The same paper also reports that two sections of TP, amino acids 287–293 and amino acids 310–324, are primarily involved in copper binding.^94^ A factor that could explain the seemingly contradictory claims is the fact that Cu^2+^ binding to TP depends both on the stoichiometry of Cu^2+^ in relation to TP, and the pH of the surrounding environment.^92^

Zn^2+^ has also been shown to accelerate the aggregation of TP.^95^ Huang *et al.* (2014) claim Zn^2+^ acts independently of TP phosphorylation.^96^ This could be possible because Hong *et al.* (1997) reported Zn^2+^ inhibits the GSK3 enzyme.^97^ Most studies have reported acceleration of aggregation at concentrations around 300 μM of Zn^2+^ (ref. 88) but concentrations as low as 10 μM (ref. 98) and as high as 500 μM are reported.^99^ Huang *et al.* (2014) studied the effects of Zn^2+^ on TP in the presence and absence of Zn^2+^ and the results suggest that Zn^2+^ clearly causes aggregation of TP *in vitro*, and even the fact that removing Zn^2+^ seems to remove the toxicity of TP.^96^

The Zn^2+^ binding site has not been clearly elucidated, though some have proposed that a cysteine residue is involved. It was demonstrated that Zn^2+^ associates with TP by coordinating with the cysteine residue of the three repeat TP constructs.^100^ Furthermore, Zn^2+^ accelerates the fibrillization of human TP by creating a “bridge” between Cys-291 and Cys-322.^101^

**Fig. 4:**
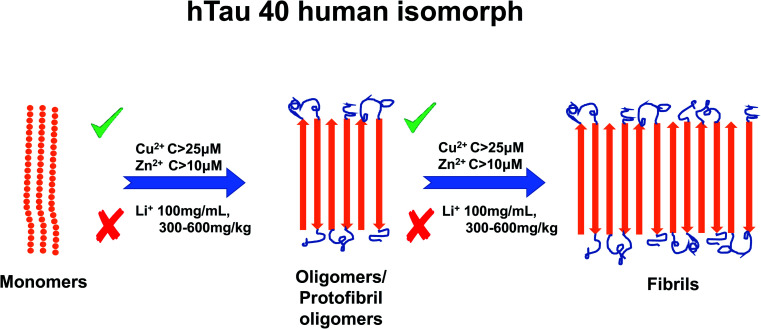
Schematic representation of metal ion concentration-dependent inhibition or acceleration of Tau protein. Zn^2+^ (concentration higher than 10 μM) and Cu^2+^ (concentration higher than 25 μM) promote aggregate formation, whereas Li^+^ (100 mg mL^−1^, 300–600 mg kg^−1^) inhibits aggregation formation. The figure has been copied and adapted with permission from Alghrably *et al.* (2019).^14^

Li^+^ presents an intriguing case as several studies have reported that it inhibits TP aggregation.^88^ Fu *et al.* (2010) reported TP phosphorylation of GSK-3β enzyme at a concentration of 100 mg mL^−1^ Li^+^,^102^ and as mentioned earlier, TP phosphorylation is a key step to TP aggregation.^80^ Su *et al.* (2004) reported a reduction in TP phosphorylation at concentrations between 300–600 mg kg^−1^.^103^ Though Li^+^ has not been as extensively studied as Cu^2+^ or Zn^2+^, one study has suggested that Li^+^ reduces Tau phosphorylation by inhibition of glycogen synthase kinase-3.^97^ For a summary of the effects of metal ion concentration on TP, refer to Fig. 4.

## 6. Amyloid-beta peptide

Like TP, Amyloid-Beta (Aβ) is also characteristic of AD. Unlike TP, Aβ has a much shorter peptide sequence; the two most common isoforms contain a total of 40 or 42 peptides only.^104^ Nevertheless, its aggregation properties are still of great importance for understanding and finding viable treatments for AD. The Aβ cascade hypothesis proposes that the deposition of Aβ is the precursor to all major stages of AD.^105^

Ha *et al.*, (2007) have shown that Aβ-40 and Aβ-42 must undergo a conformational change before aggregation of this protein can start.^106^ Novo *et al.* (2018) studied the effects of Aβ-42 concentrations on Aβ-42 aggregation.^107^ The relationship is not linear, but rather sigmoidal in nature. They discovered that aggregation of Aβ-42 does not occur until Aβ-42 has reached a critical aggregation concentration of 90 nM. Even at this critical aggregation concentration, only a small percentage (approximately 10%) of Aβ-42 proteins will aggregate, and most Aβ-42 proteins will not aggregate until the concentration of Aβ-42 proteins is considerably higher than 90 nM.^107^

The effect of metal ion concentrations on Aβ has also been studied extensively,^88^ and generally must be considered on a case by case basis since the type of ion and its relative amount (stoichiometry) to Aβ can have enormous implications.^108^ At concentrations of 100 μM, Cu^2+^ and Zn^2+^ cause amorphous aggregation of Aβ-42. The presence of Cu^2+^, Zn^2+^ and Fe^3+^ at concentrations of 100 μM increases the volume of aggregated Aβ-42 by a significant percentage.^106^ The effects of Hg^2+^ and Pb^2+^ at concentrations of 0.25 μM, 2.5 μM, 25 μM, and 250 μM were studied, whereby the amount of Aβ-42 also increased.^109^ Although much less research has been carried out on the impact of Al^3+^, it has been shown to accelerate the aggregation of Aβ-40.^110^

In recent years, several efforts have been undertaken to determine how metal ions bind to Aβ, though this is a challenging task because Aβ can change its shape depending on the electronic and structural properties of the binding metal ion.^111^ Nevertheless, sites for Cu^2+^ binding to Aβ have been proposed. The most commonly proposed site for Cu^2+^ binding includes the imidazole ring of a histidine residues at position 6, 13 and 14, the N-terminal amine group, and an adjacent CO functional group from the Asp1–Ala2 peptide bond.^112^ Many articles argue that the imidazole ring of the histidine residue is required for Cu^2+^ binding to Aβ, and that the Cu^2+^ binding mechanism is distinct from the other binding mechanisms of Zn^2+^, Fe^3+^, and Al^3+^.^113,114^ Cu^2+^ is also proposed to control Aβ-42 aggregation at submolar concentrations by forming dityrosine linkages between Aβ-42 monomers.^115^

The binding mechanism of Zn^2+^ on Aβ also deserves some recognition. Zn^2+^ binds in the same hydrophilic region (Asp1–Lys16) as Cu^2+^ (ref. 116) although, perhaps paradoxically, Zn^2+^ increases the total amount of exposed hydrophobic parts on Aβ, whereas Cu^2+^ decreases it. Perhaps even more striking is the fact that Zn^2+^ diminishes the lag time that Aβ experiences upon aggregation, even at small concentrations (5 μM), while Cu^2+^ at similar concentrations increases the lag time to above 60 hours.^113^ Several have proposed Zn^2+^ adopts a tetrahedral coordination, where like its Cu^2+^ counterpart, associates with histidine residues on Aβ.^116^

**Fig. 5:**
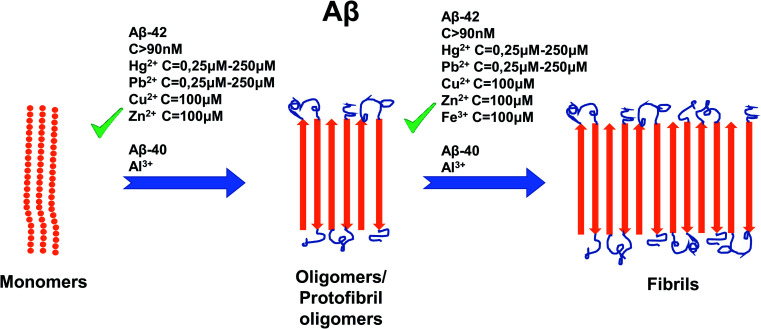
Schematic representation of metal ion concentration-dependent inhibition or acceleration of Aβ. Concentrations of Aβ-42 (more than 90 nM), Hg^2+^ (0, 25–250 μM), Pb^2+^ (0, 25–250 μM), Zn^2+^ (100 mM), Cu^2+^ (100 μM) and Fe^3+^ (100 μM) promote the formation of aggregates, whereas for Aβ-42 only, Al^3+^ favors formation of aggregates. The figure has been copied and adapted with permission from Alghrably *et al.* (2019).^14^

Al^3+^ presents an interesting case, as in AD patients its concentration is about 1.6 times higher than that of normal people.^117^ It was reported that toxic amyloid chambers form when Al^3+^ and Aβ oligomers aggregate in sync with each other.^117^ This finding may lead future researchers to discover the true binding site of Al^3+^ to Aβ. Like Cu^2+^ and Zn^2+^, it has a distinct, measurable effect on Aβ aggregation^113^ and therefore, is likely to have its own unique mechanism of binding of Aβ. For a summary of the effects of metal ion concentration on Aβ, refer to Fig. 5.

Other metal ions such as Mn^2+^, Mg^2+^ and Cd^2+^ and their effects on the aggregation of Aβ have also been examined. As for the case of TP, some metal ions cause acceleration of aggregation of Aβ-40 or Aβ-42, and others cause inhibition.^88^ Understanding the precise relationship between concentrations of metal ions and aggregation of Aβ-40 or Aβ-42 will provide interesting research opportunities for the scientific community, as well as helping to find a viable treatment for AD patients.

## 7. Polyglutamine: Huntington's disease

Polyglutamine (PolyQ) is more complicated than some of the previously presented proteins in this review. PolyQ is associated with at least nine separate diseases, the most prominent one being Huntington's disease (HD).^118^ Since the most studied disease related to PolyQ is HD, the remainder of this section will focus on the effect of metal ions related to HD, and its protein, huntingtin 1.

It is known that PolyQ only becomes toxic in HD only after extending beyond a pathological length^119^ and that its length is crucial to its aggregation properties.^120^ PolyQ's aggregation process is distinct from that of other proteins discussed in this review,^120^ but unlike the other proteins discussed here, its aggregation process is less well understood. As for the factors inducing aggregation of PolyQ in HD, there is a scarcity of information. Currently, it was confirmed that the length of glutamine repeats affects the aggregation process. Yushchenko *et al.* (2018) demonstrated that repeats of 11 glutamines are not sufficient to cause PolyQ aggregation, however longer sequences of 38 and 56 tend to stimulate aggregation, with 56 repeats having higher aggregation kinetics than 38.^121^ In terms of metal ions, it was found that copper binds to the first 171 residues on the N-terminal region of huntingtin 1, which contains PolyQ repeats and promotes aggregation of huntingtin 1.^122^ His82 and His98 were identified as crucial for copper binding. However, there is a lack of information as to whether the length of glutamine residues affects the binding of copper.^122^ Xiao *et al.* (2013) also reports a histidine residues being involved in binding, and also suggest that Cu^2+^ bind to the residue Met8.^123^ The same authors report that HD arises from a combinatory toxicity of PolyQ and Cu^2+^, that is, Cu^2+^ is actually required to cause HD.^123^ Interestingly enough, zebrafish that lack the huntingtin protein exhibit sizeable defects in iron utilization and development, meaning that huntingtin (PolyQ) may play a role in iron pathways.^124^

Kar. *et al.* (2011) propose that aggregation proceeds *via* a nucleus centered approach, although several other aggregation mechanisms have been proposed.^118,120^ A beta sheet is likely involved^125^ and PolyQ only aggregates after reaching a critical aggregation concentration of 3 μM.^120^ Even so, these results are suggestive at best, and clearly indicate the need for additional studies specifically on HD, its protein huntingtin 1 and the PolyQ repeats it contains.

## 8. Conclusion and future outlook

There is significant ongoing effort to understand the relationship between metal ions and their effect on protein aggregation. Protein aggregation and misfolding are recurrent in many neurodegenerative diseases (*i.e.* Parkinson's, Alzheimer's, *etc.*).^126^ The relationship between the metal ions and protein aggregation is difficult to describe precisely because even a slight change of the external environment (pH, metal ion/protein concentration, *etc.*) can disrupt the fragile equilibrium state of the functional protein.^126^ The disorderliness of Tau and α-synuclein, for example, is context specific,^127^ including in the presence of metal ions.

Some studies have sought to create experiments that might explain more clearly how some proteins aggregate (specifically, TP and α-synuclein) aggregate,^128–132^ and one paper even claims to have invented a simple and reproducible method for monitoring the aggregation of α-synuclein aggregation^133^ in a plate-reader based assay. The protocol utilizes Thioflavin T (ThT) fluorescence to measure the kinetics of the aggregation of α-synuclein.^133^ Protocols such as this could be developed to explain the seemingly obscure relationship between protein aggregation and metal ion concentration. Understanding how protein aggregation works has led some scientists to develop anti-aggregation drugs against TP and α-synuclein.^134–136^ More systematic experiments designed to clarify this relationship are vital, as they may provide the groundwork to produce better therapeutics. Therefore, further research with more rigorous and detailed studies are necessary to definitively uncover the relationship between metal ions and their effects on the aggregation of proteins, with a particular emphasis on their concentrations and relative ratios.

This detailed knowledge about the link between protein and metal ion concentration and the amount of aggregation would give us a necessary level of understanding of the biochemical processes behind the complex, multi-step aggregation process that would allow us to design better inhibitors (ultimately more efficient and commercially available drugs) of the aggregates formation at the early soluble state. It may result in efficient targeting of the early state of the aggregation process in which smaller and soluble aggregates are formed as a result of the association of β-sheet motifs to each other.^31,137^

It is an obvious fact that the surrounding environment of the protein must be also considered in these future studies, and not just the proteins in isolation with metal ions. Amylin aggregation, for example, is strongly pH dependent with its two protonable sites at His18 and at the N-terminus.^138^ α-synuclein fibrils can form under several different solution conditions, but only a handful of these conditions lead to rapid multiplication of α-synuclein fibrils. Clearly, the solution conditions determine the relative importance.^62^ Designing compounds to successfully inhibit amylin aggregation will require a good amount of strategy because inhibition of amylin aggregation process may not automatically delete its cytotoxicity to islet β-cells.^139^

Based on the currently available scientific literature, we may speculate about possible aggregation mechanisms of proteins. It was suggested that α-synuclein may aggregate more quickly *via* oligomer–oligomer interactions than *via* monomer–monomer interactions.^140^ Another study corroborates this idea by suggesting that seeding of monomers of α-synuclein is not sufficient to cause α-synuclein aggregation, but rather, exhibits prion-like spreading.^141^ It is reasonable to speculate that other proteins (TP, amylin, α-synuclein, *etc.*) may aggregate *via* oligomer-induced cellular stress, rather than through the precise coordination of the monomers of these proteins.

Metal ions concentrations are only one of several factors that strongly influence the increase or decrease in protein aggregation. Given the current gaps in knowledge relating to this specific factor, and given the potential knowledge that understanding the effect of metal ion concentrations on protein aggregation can provide researchers and scientists regarding the subject of protein aggregation, there is a clear need for further investigation of this topic for the advancement of future therapeutics of protein aggregation related diseases.

## Conflicts of interest

There are no conflicts to declare.

## Supplementary Material
